# Bioengineering studies and pathway modeling of the heterologous biosynthesis of tetrahydrocannabinolic acid in yeast

**DOI:** 10.1007/s00253-020-10798-3

**Published:** 2020-10-12

**Authors:** Fabian Thomas, Christina Schmidt, Oliver Kayser

**Affiliations:** TU Dortmund University, Technical Biochemistry, Emil-Figge-Strasse 66, 44227 Dortmund, Germany

**Keywords:** *Cannabis sativa*, Cannabinoids, Tetrahydrocannabinol, Cannabidiol, Synthetic biology, Bioengineering, Natural Product Biotechnology, NphB, CsPT

## Abstract

Heterologous biosynthesis of tetrahydrocannabinolic acid (THCA) in yeast is a biotechnological process in Natural Product Biotechnology that was recently introduced. Based on heterologous genes from *Cannabis sativa* and *Streptomyces* spp. cloned into *Saccharomyces cerevisiae*, the heterologous biosynthesis was fully embedded as a proof of concept. Low titer and insufficient biocatalytic rate of most enzymes require systematic optimization of recombinant catalyst by protein engineering and consequent C-flux improvement of the yeast chassis for sufficient precursor (acetyl-CoA), energy (ATP), and NADH delivery. In this review basic principles of in silico analysis of anabolic pathways towards olivetolic acid (OA) and cannabigerolic acid (CBGA) are elucidated and discussed to identify metabolic bottlenecks. Based on own experimental results, yeasts are discussed as potential platform organisms to be introduced as potential cannabinoid biofactories. Especially feeding strategies and limitations in the committed mevalonate and olivetolic acid pathways are in focus of in silico and experimental studies to validate the scientific and commercial potential as a realistic alternative to the plant *Cannabis sativa*.

Key points

*• First time critical review of the heterologous process for recombinant THCA/CBDA production and critical review of bottlenecks and limitations for a bioengineered technical process*

*• Integrative approach of protein engineering, systems biotechnology, and biochemistry of yeast physiology and biosynthetic cannabinoid enzymes*

*• Comparison of NphB and CsPT aromatic prenyltransferases as rate-limiting catalytic steps towards cannabinoids in yeast as platform organisms*

**Figure:**
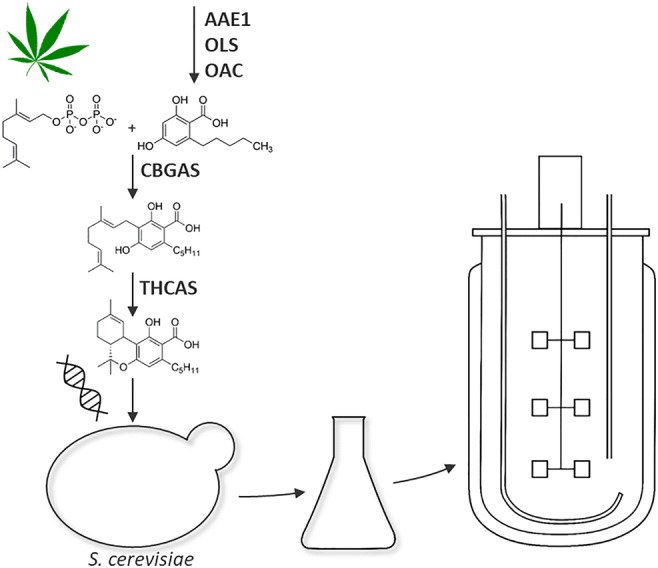
Graphical abstract

Graphical abstract

## Introduction

Since the legalization of cannabis products for medicinal use, cannabinoids like tetrahydrocannabinol (THC) and cannabidiol (CBD) get attraction as for direct use or as potential drug candidates for various diseases. Besides isolation from plant material, the biotechnological production of cannabinoids like THC and CBD is an exciting alternative. Over the last years, the genetic blueprint of THC and CBD biosynthesis is understood, and genes have been functionally expressed in various microorganisms as documented in scientific papers. So far, no report has been communicated showing scaled up biosynthesis and how an industrial process must be designed to allow feasible economic production in a bioreactor. In this review, we take for the first time the endeavor to analyze basic biological parameters and to develop concepts and strategies for an engineered process to identify limitations, bottlenecks, and opportunities.

**Fig. 1:**
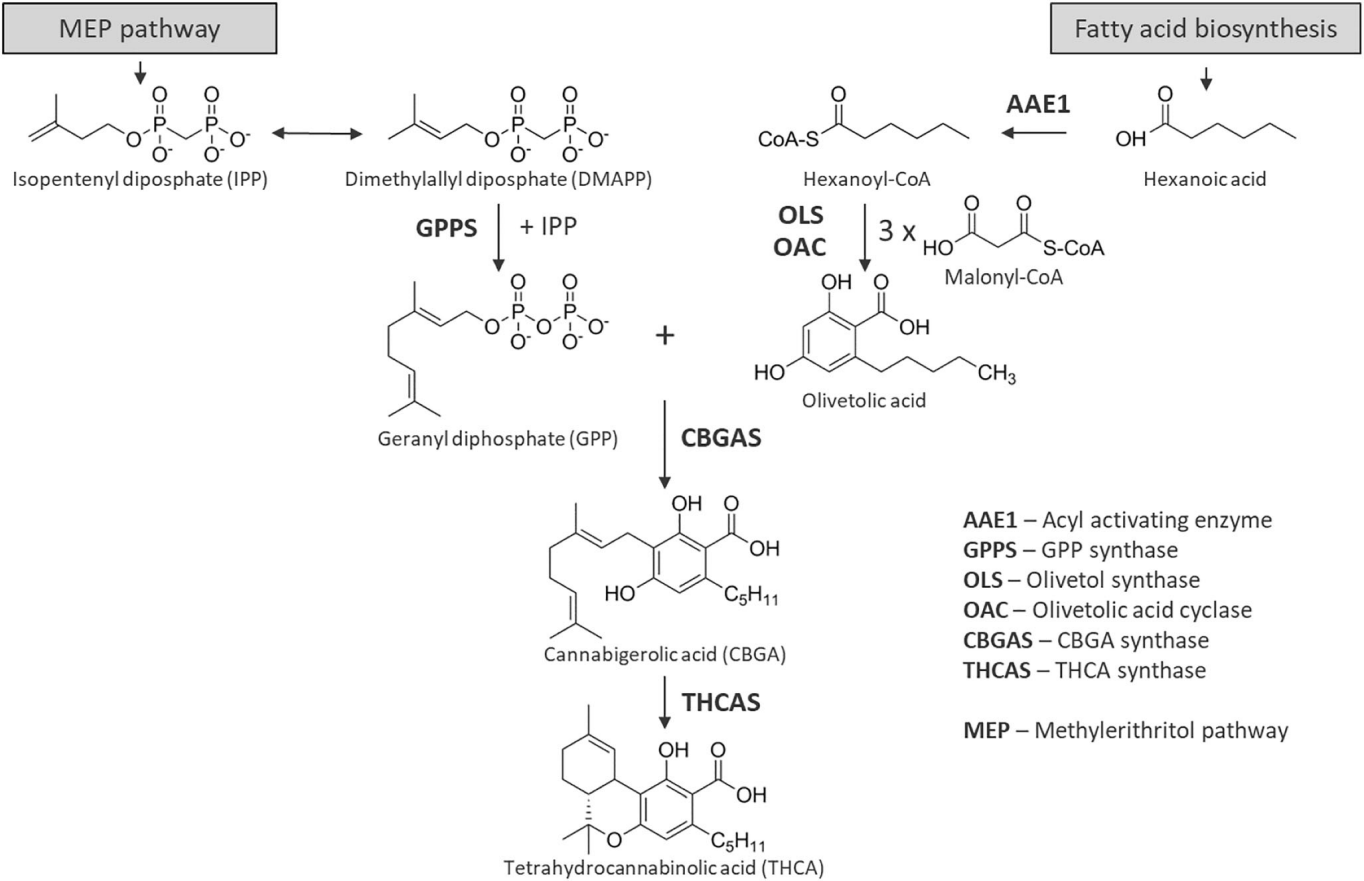
Tetrahydrocannabinolic acid (THCA) biosynthesic pathway in *C. sativa* L.. A total of six enzymes (AAE1, GPPS, OLS, OAC, CBGAS, THCAS) form THCA from isopentenyl diphosphate (IPP) and dimethylallylphosphate (DMAPP) synthesized in the MEP pathway, as well as hexanoic acid provided by the fatty acid biosynthesis

Cannabinoids seem to be a unique class of secondary natural products limited to *Cannabis sativa* L. In recent time, prenylated olivetolic acid derivatives and other structurally related prenylated phenolics have also been identified in various genus and species like *Helichrysum umbraculigerum* Less. (Pollastro et al. 2017) or the liverwort *Radula marginata* Taylor (Asakawa et al. 1991; Nagashima and Asakawa 2011). The biosynthesis of tetrahydrocannabinolic acid (THCA-C5) and its precursors derived from the mevalonate and olivetolic acid pathway, as we understand today. Without going into the details of molecular biology, genetics and spatial resolution of biosynthesis (Fig. 1) (Degenhardt et al. 2017), all committed biosynthetic enzymes on the way to tetrahadrocannabinolic acid, but also cannabidiolic acid and cannabichromenic acid, share the precursor cannabigerolic acid (CBGA-C5). Furthermore, all conversion products have the same mass and differ only structurally. Recently, the full recombinant biosynthesis of THCA was published by Luo et al. (2019) in *Saccharomyces cerevisiae* as a heterologous host. Here, the native mevalonate pathway in *S. cerevisiae* and a multi-organism-derived hexanoyl-CoA biosynthetic pathway have been implemented as well. With a modified primary metabolism for delivery of the essential precursors IPP/DMAPP (isopentenyl diphosphate/dimethylallyl diphosphate) and pyruvate, the upstream biosynthesis towards olivetolic acid was accessible. Especially pyruvate plays an important role as a central metabolite for the biosynthesis of hexanoic acid and as a decarboxylated starter (acetyl-CoA) for the mevalonate pathway. By the introduction of the genes to encode olivetolic acid cyclase (OAC) and olivetol synthase /(OLS) for the biosynthesis of olivetolic acid, and the integration of corresponding cannabinoid synthases like CBGAS and THCAS, a new biological system was engineered for the production of natural and unnatural cannabinoids.

With all respect to the excellent work in synthetic biochemistry and the full assembly of the pathway towards THCA-C5, unfortunately, the production yield of CBGA-C5 (7.2 μg L^−1^), THCA-C5 (8 mg L^−1^), and CBDA-C5 (4.4 μg L^−1^) are low (Luo et al. 2019). Future challenges and needs are to gain a better fundamental understanding of the mode of catalysis in yeast to increase production titer in *S. cerevisiae* drastically. In this review, we focus on the potential use of computational and mathematical systems biotechnology to develop an in silico model for the heterologous biosynthesis of THCA in *S. cerevisiae*. This yeast is an industrial GRAS organism and used for its high production of IPP and DMAPP because of an engineered mevalonate pathway. By in silico modeling, the prediction and optimization of the heterologous biosynthesis of THCA are desirable to gain fundamental insight into the primary metabolism as a metabolic basis to tune yeast physiology and to have quantitative data on theoretical energy consumption, as well on ATP, NADPH, and NADH usage. In this review, we want to give a hypothetical example of the production of THCA in a fermenter. With all anticipated limitations, we are aware of and we try to explore the industrial potential as a prototype.

### *Saccharomyces cerevisiae* as a model organism

Systems biotechnological studies are conducted with *S. cerevisiae* as a model organism. This yeast is used in the industry widely because of its GRAS status, easy to handle physiological properties, and wide knowledge in genomics, proteomics, and other related fields of “OMICS.” It is very attractive for the THCA production because of high yield terpenoid biosynthesis and delivery of IPP/DMAPP (Ro et al. 2006; Keasling 2012). Tools for genetic manipulation are also available with this organism, and genetic manipulation is easy to conduct (Krivoruchko and Nielsen 2015). For the bioprocess design, it is essential to consider *S. cerevisiae* as yeast that is acidophil and grows best under acidic conditions around pH 5.5. *S. cerevisiae* is a facultative anaerobe, i.e., it can gain energy through breathing and fermentation. Under oxygen consumption, preferably glucose as a carbon source is entirely reduced to ATP and CO_2_, while under anaerobic conditions, glucose is fermented to ethanol generating lower amounts of ATP.

For the biosynthesis and production of cannabinoids, *S. cerevisiae* has more advantages: first, a higher biocatalytic activity being explained by low concentration of misfolded biosynthetic proteins and absence of inactive inclusion bodies; second, glycosylation of the THCAS resulting in improved solubility correlated with increased biocatalytic rate; and last, the ability to secrete cannabinoids to minimize the risk of self-toxification and to allow easier downstreaming and isolation.

In our study, we have not considered other sugars than glucose as feed. However, other carbohydrates, as well as ethanol and fatty acids, may also be accepted as carbon sources for metabolism. However, glucose is a standard carbon source, and its metabolization is easy to trace why the stoichiometric calculation is simple. It must be noted that glucose metabolization in yeast is critical regarding anaerobic ethanol production and at high aerobic concentration due to respiratory inhibition known as the Crabtree effect (De Deken 1966). Both must be considered in all metabolic models applied. The fermentation of glucose to ethanol results in decreased cell growth due to a lack of ATP for biomass production. Substrate deficiency leads to degradation of previously synthesized ethanol. This reintroduction into the C-flux is an opportunity to use the ethanol produced through the Crabtree effect as carbon source buffer.

### Toxicity of tetrahydrocannabinol

Like other neutral cannabinoids, tetrahydrocannabinol (THC-C5) is a hydrophobic compound with very low solubility in water (< 10 mg/L) and has low toxicity to humans if administered. So far, no data are available for the cytotoxic effect of THC on *S. cerevisiae*. In our unpublished studies, we identified for THC the EC_50_ value of approximately 360 mg/L in direct incubation assays. This concentration is sufficiently high to be tolerated in almost all relevant batch fermentations. This toxicity range is also essential to estimate the production window between toxicity and maximum catabolic rate. THCA synthase catalyzes the reaction from cannabigerolic acid to tetrahydrocannabinolic acid along with an equimolar production of hydrogen peroxide (Sirikantaramas et al. 2007). Hydrogen peroxide has cytotoxic effects on yeast cells (Zirpel et al. 2015). However, *S. cerevisiae* has developed catalase enzymes as a defense mechanism to increase the tolerance of hydrogen peroxide (Izawa et al. 1996). It is unclear if THCA or hydrogen peroxide causes the toxic effect on genetically modified yeast cells (Zirpel et al. 2015). Our experiments show that experimental heterologous production of THCA in *S. cerevisiae* is limited to a maximum concentration of 280 mg/L and even below the EC_50_ value of direct incubation. We must consider the concentration of yeast maximum production as too low and safe in a controlled bioprocess to avoid severe impairment. Our in silico model does not contain the limitation of the cell activity. Hence, the model is not accurate if THCA concentrations exceed unlikely the toxic threshold.

## Biosynthetic enzymes

### Olivetol synthase and olivetolic acid cyclase

In *Cannabis sativa* L., olivetolic acid, as one of the precursors for the production of CBGA, is formed with three molecules of malonyl-CoA by a type III polyketide synthase (olivetol synthase, OLS) (Taura et al. 2009) in combination with a polyketide cyclase (olivetolic acid cyclase, OAC) from hexanoyl-CoA (Gagne et al. 2012) (Table 1). The presence of OAC is crucial for the production of olivetolic acid, as OLS alone is only able to produce olivetol and the two α-pyrone pentyl diacetic acid lactone (PDAL) and hexanoyl triacetic acid lactone (HTAL) by-products (Gagne et al. 2012). OLS is proposed to be a homodimeric enzyme of approximately 89 kDa, with each part consisting of a 385 amino acid polypeptide (Taura et al. 2009). OAC is reported as an α + β barrel (DABB) protein composed of 101 amino acids and catalyzes the C2–C7 aldol condensation of the intermediate 3,5,7-trioxododecanyl-CoA maintaining the carboxylate moiety to form olivetolic acid. Functional expression of both enzymes originated from *C. sativa* L. as well as the production of OA in *S. cerevisiae* has been reported (Gagne et al. 2012; Luo et al. 2019). Site-directed mutagenesis studies revealed a variant of OAC (Y27F) with a 162 % relative activity compared with the wild-type enzyme (Yang et al. 2016).

**Table 1:** Summary of the chemical and physiological properties of the main enzymes of the later cannabinoid biosynthesis

Properties	THCAS	CBDAS	CBCAS	CBGAS	NphB	OAC	OLS
Accession no.	AB057805	AB292682	n.a.	n.a.	AB187169	AFN42527.1	AB164375
EC no	1.21.3.7	1.21.3.8	1.3.3.-	2.5.1.102	2.5.1.39	4.4.1.26	2.3.1.206
Location	Cytosol	Membrane bound in chloroplast	n.d.	Membrane bound	Cytosol	Cytosol	Cytosol
Source	*C. sativa*	*C. sativa*	*C. sativa*	*C. sativa*	*Streptomyces* spp.	*C. sativa*	*C. sativa*
MW	59 kDa	62 kDa	n.d.	74 kDa	34 kDa	23 kDa	45 kDa
pH optimum	5.5–6.0	5.0	n.d.	7.0	n.d.	n.d.	5.5
Temp. optimum	50 °C	n.d.	n.d.	30 °C	n.d.	n.d.	30 °C
Structural data	3VTE	n.a.	n.a.	n.a.	1ZCW	5B08 (OAC apo) 5B09 (OACOA binary complex)	6GW3

THCAS: tetrahydrocannabinolic acid synthase, CBDAS: cannabidiolic acid synthase, CBCAS: cannabichromenic acid synthase, CBGAS: cannabigerolic acid synthase, NphB: prenyltransferase from *Streptomyces* sp. strain CL190, OAC: Olivetolic acid cyclase, OLS: olivetolic synthase, *n.a.* not applicable, *n.d.* not determined

### Cannabigerolic acid synthase and NphB

Cannabigerolic acid synthase (CBGAS) and NphB (Table 1) are both described as aromatic C-prenyltransferases. The aromatic prenyltransferase cannabigerolic acid synthase (CBGAS) is responsible for the C–C prenylation of olivetolic acid (OA) by geranyl diphosphate (GPP) to form a cannabigerolic acid in *C. sativa* according to the Friedel-Crafts alkylation (Fellermeier and Zenk 1998; Fellermeier et al. 2001). CBGAS is presumed to be an integral membrane protein, although protein activity was found in soluble fractions only (Fellermeier and Zenk 1998). However, Luo et al. (2019) used the prenyltransferase CsPT4 from *C. sativa* to produce CBGA and finally reconstruct the whole pathway for the biosynthesis of THCA in *S. cerevisiae* and claimed much higher catalytic turnover.

In contrast to CBGAS, NphB is a soluble catalyst. The prenyltransferase NphB from *Streptomyces sp.* strain CL190 is a promising alternative to the membrane-bound CBGAS. Heterologous *NphB* expression was shown in the yeasts *K. phaffii* and *S. cerevisiae*, and NphB can produce CBGA from GPP and OA in principle, but the *O*-prenylated product 2-*O*-geranyl olivetolic acid (2-*O*-GOA) was mainly detected (Zirpel et al. 2017). Due to the nucleophilic nature of the aromatic hydroxyl groups in the positions C4 and C6, *O*-prenylation as a side reaction leads to unwanted metabolites. However, protein engineering of NphB revealed a variant (Y288A/G286S), which produces the desired product CBGA with 1000-fold increased specific activity (k_cat_), about 175-fold increased activity but in a cell-free system only (Valliere et al. 2019). 

### Tetrahydrocannabinolic acid synthase

Tetrahydrocannabinolic acid synthase (THCAS) catalyzes the oxidative cyclization of CBGA, which represents the final enzymatic step in the biosynthesis of THCA (Table 1). The enzyme consists of 545 amino acids and has a monomeric structure divided into two domains by a flavin adenine dinucleotide (FAD) binding pocket, in which the FAD molecule is covalently bound to the enzyme at positions H114 and C176 (Shoyama et al. 2012). Covalent binding of the FAD molecule is essential for the activity of THCA synthase (Sirikantaramas et al. 2004; Zirpel et al. 2018b). A disulfide bond is formed between C37 and C99 of domain I, and at least six N-glycosylation sites were detected. Y484 is essential for the catalytic activity of THCAS, and the amino acids H292 and Y417 are suggested to be important for substrate stabilization or binding (Shoyama et al. 2012). Protein engineering of THCAS resulted in a 2-fold increased activity of THCAS variant N89Q/N499Q and a 1.7-fold increased activity of THCAS variant H494C/R532C, aiming to add a disulfide bond (Zirpel et al. 2018b).

## Modeling of the heterologous biosynthesis

The heterologous biosynthesis of tetrahydrocannabinolic acid (THCA-C5) requires a full redesign of yeast metabolism and physiological adaptation. The stepwise bioengineering of enzymes or whole pathways in vivo is time-consuming and far from trivial. Here, we seek to establish an in silico platform for THCA-C5 biosynthesis in an extended kinetic model based on recent literature and our expertise.

In the first step of our in silico design, the heterologous pathway is constructed as an unbranched linear metabolic pathway in *S. cerevisiae.* From the generation of simple building blocks like acetyl-CoA towards THCA-C5 synthesis, the pathway is implemented concerning natural precursors. Throughout, energy and cofactor supply, as well as their respective usages, are tracked, starting with glycolysis over the citric acid cycle up to the cannabinoid pathway. In general, we assumed glucose uptake and metabolization via glycolysis to be the central pathway for the delivery of acetyl-CoA as a C2 building block for the mevalonate and fatty acid biosynthesis. Without consideration of compartmentalization in *S. cerevisiae*, direct cytosolic biotransformation of acetyl-CoA to olivetolic acid is assumed. Stepwise, we will discuss and evaluate significant aspects of the implementation of model components and the metabolic engineering strategies leading to their inclusion for all mentioned pathways.

### Modeling theory

We constructed an extended kinetic model based on available kinetic parameters found in common enzyme reaction kinetics databases like BRENDA (Brenda 2019; Jeske et al. 2019) or SABIO-RK (Wittig et al. 2012; SABIO 2019). All data was transferred to a model raised in MATLAB® *version 9.4* with the SimBiology extension (The MathWorks 2017). Where kinetic data was unavailable or scarce for *S. cerevisiae*, enzyme data of closely related species were considered and evaluated first before adding the parameters to the model. For most reactions, we used simple Michaelis-Menten kinetics and only implemented complex multi-substrate enzyme kinetics like ordered bi-bi kinetics, where one substrate could not be regarded as in excess compared with the other. On several occasions, we modified existing kinetic data to better reflect recent experimental finds of our workgroup. Although COPASI has a broad range of interesting and sophisticated analytical tools, some of them like sensitivity analysis is lacking to be correct to model kinetics at non-steady-state levels. Furthermore, the metabolic model of THC biosynthesis in yeast contains metabolic branches, and THCA accumulates over time; it is not possible to create a real steady-state model. Therefore, COPASI finds its limitation to perform a sensitivity analysis for our purpose. SBML files were exported from COPASI to SimBiology add-on of MATLAB® to overcome this problem.

### General model implementation and scope

We modeled the THCA-C5 production starting from a simple glucose feed, passing glycolysis and the citric acid cycle to simulate energy supply. Product formation would then occur after simplified olivetolic acid, and mevalonate pathways provided olivetolic acid and geranyl pyrophosphate, respectively. Compartmentalization was mostly ignored for the sake of simplicity; whenever applicable, formation rates of intermediate species factor in their transport to the compartment they are needed in. This then firstly results in a yeast cell compartment, containing all pathway reactions towards THCA, and secondly a medium compartment, where nutrients, like glucose, are located. They are connected through their respective uptake rates; the glucose uptake, for instance, was modeled after Teusink et al. (2000).

The glycolysis is satisfyingly well understood; we decided however to implement the pentose phosphate pathway as a black box as suggested by Chen et al. (2012). In the finished model, a typical glucose concentration of 111 mM is completely metabolized after 20.8 h, which is in accordance with our in vivo experiments (data not shown). For the citrate cycle, the typical enzymes are implemented without significant modifications; only a separate mitochondrial acetyl-CoA pool beside the cytosolic pool is tracked through a different species.

Increasing the acetyl-CoA is metabolic challenge. It is widely known as text book knowledge that in yeast, cytosolic and mitochondrial acetyl-CoA pools are strictly separated pools, and mitochondrial acetyl-CoA cannot be transported into the cytosol. To simulate the acetyl-CoA pool as accurate and expressive as possible in the cytosol, we use the ATP citrate lyase, a modified pyruvate dehydrogenase bypass and a glucose-regulated ADH2 that enhances acetaldehyde production. These strategies are discussed in greater detail below. The ethanol generation through the Crabtree effect is simulated in an isolated species to reduce complexity. Furthermore, the naturally biosynthesized polyketide and GPP building blocks are insufficient for a powerful THCA producer, which is why their respective pathways need to be altered significantly. Both are discussed in dedicated sections below, where we elaborate on suitable enzymes to add to the in silico model.

## Refinement of the in silico pathway through current bioengineering strategies

### Acetyl-CoA levels

Acetyl-CoA is the committed precursor for both the mevalonate and olivetolic acid pathway to GPP and olivetolic acid. Regulation and increase of the acetyl-CoA or short C2 pool are essential for catalytic turnover and yield. Consequently, intracellular levels of acetyl-CoA, as well as its delivery from the primary pathway in *S. cerevisiae* to the heterologous cannabinoid biosynthesis, constitute a significant concern in any successful bioengineering strategy.

As an alternative to the pyruvate dehydrogenase (PDH) route of pyruvate to acetyl-CoA, the PDH bypass is favored by *S. cerevisiae* wild type during fermentative metabolism to supply cytoplasmatic pathways (MVA, PK) with acetyl-CoA (Remize et al. 2000). The increased demand of cytosolic acetyl-CoA for the production of monoterpenes immediately suggests an overexpression of the PDH bypass genes coding for pyruvate decarboxylase, acetaldehyde dehydrogenase, and acetyl-CoA synthetase (Shiba et al. 2007). However, it was shown that along with acetyl-CoA, also acetate levels were increased, which in turn induced oxidative stress in the cells. Overall, the acetate-driven drop in cell viability is not worth the additional cytosolic acetyl-CoA in *S. cerevisiae* (Semchyshyn et al. 2011b; Semchyshyn et al. 2011a). A more promising approach is replacing acetaldehyde dehydrogenase (ADH) as well as acetyl-CoA synthetase with aldehyde dehydrogenase acylating (ADA) from *Dickeya zeae*. This simple optimization features an overall higher specific activity, the prevention of acetate formation, and most importantly demands less energy. The ATP cost of GPP decreases 12-fold through this change (Meadows et al. 2016). Cytosolic acetyl-CoA levels can be increased further by providing more acetaldehyde for ADA to convert. The key idea is to utilize the ethanol generated by aerobic cultivation on glucose. By overexpression of the yeast *ADH2* gene under the control of the strong glucose-regulated promoter of pHXT7, the yeast is able to convert ethanol, produced by Adh1p, back to acetaldehyde. Through ADA the cytosolic acetyl-CoA concentration will rise further.

In addition to the aforementioned strategies it is highly beneficial to direct the acetyl-CoA cytosolic flux away from non-essential pathways and towards cannabinoid biosynthesis. In this regard, the glyoxylate cycle, which has been shown to be active even when glucose is present in the medium, can be targeted. Both peroxisomal citrate synthase and cytosolic malate synthase effectively decrease the cytosolic acetyl-CoA pool. Deletion of the respective genes CIT2 and MLS1 has been suggested to reduce the consumption of cytosolic acetyl-CoA, which in turn can then be utilized for the mevalonate pathway and the fatty acid biosynthesis required for cannabinoid production (Chen et al. 2012; Chen et al. 2013).

### Olivetolic acid production

The efficiency of the heterologous cannabinoid biosynthesis depends on the olivetolic acid supply, which is, together with GPP, consumed in equimolar amounts by NphB forming CBGA. We observe in initial modeling approaches that the olivetolic acid pool is significantly lower than the GPP pool, leading to limited THCA production due to non-sufficient CBGA availability. To overcome this severe problem, different strategies to enhance olivetolic acid production were evaluated.

The metabolic delivery of essential precursors, like malonyl-CoA, is a key challenge for upregulation OA production. For each mole of olivetolic acid, three equivalents of malonyl-CoA are required, which are provided by the carboxylation of acetyl-CoA. As the rate-limiting step in lipogenesis (Yu et al. 2016), slow malonyl-CoA formation correlates with poor olivetolic acid yields. To overcome this problem, the authors have proposed several modifications to address this issue (Carvalho et al. 2017; Zirpel et al. 2017). A most promising approach is the overexpression of the *ACC1* gene encoding acetyl-CoA carboxylase to improve malonyl-CoA levels 2-fold (Runguphan and Keasling 2014). Metabolic engineering of *S. cerevisiae* for the production of fatty acid-derived biofuels and chemicals by Runguphan and Keasling (2014) gives an excellent example of this metabolic bottleneck.

Furthermore, the overall activity of acetyl-CoA-carboxylase was improved 3-fold after mutating two phosphorylation sites of ACC1 (Lian et al. 2018). The mutated enzyme was no longer recognized or inactivated through phosphorylation by protein kinase SNF1. These significant changes were considered and implemented in our kinetic model to improve it significantly. In a yeast cell, a steady supply of hexanoyl-CoA is furthermore needed along with malonyl-CoA to form olivetolic acid. A closer look at the fatty acid biosynthesis of *S. cerevisiae* reveals that the delivery of hexanoyl-CoA through the native pathway is rather weak. The limited biosynthesis of hexanoic acid (caproic acid) can explain this by the cytosolic multi-enzyme complex FAS1 that has a much higher activity for the production of long-chain fatty acids (Sheng and Feng 2015). Numerous bioengineering strategies have been proposed to replace FAS1 with recombinant enzymes or enzyme variants to increase the short-chain fatty acid titers (Leber and Da Silva 2014; Gajewski et al. 2017). Although these strategies are excellent to improve the hexanoic acid level by two magnitudes in initial tests of our kinetic model, still the biosynthetic pathway is limited by slow catalyzing enzymes and needs further improvements.

**Fig. 2:**
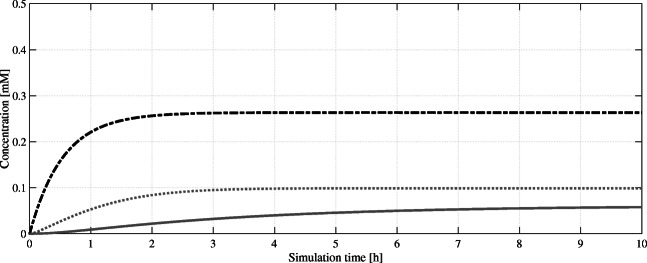
Simulated intracellular concentrations of olivetolic acid pathway intermediates over a simulation time of 10 h. Hexanoic acid (HA, ), hexanoyl-CoA (HCoA, ), and olivetolic acid (OA, ) reach an intracellular non-toxic steady state

The model performed much better with a simple feeding strategy of hexanoic acid (HA) in supplemented media. Feeding of HA will avoid physiological impairment resulting from a lack of long-chain fatty acids affecting normal cell growth by genetic knock out mutations. The feasibility of this feeding approach was evaluated in experiments since hexanoic acid acts as cytotoxin causing membrane stress in yeast (Liu et al. 2013). Cells of *S. cerevisiae* can be cultivated with up to 1 mM hexanoic acid without showing altered growth behavior (unpublished data by the authors). It is clear from the model that even if the simulated cells are provided continuously with 1 mM hexanoic acid, the intracellular concentration shown in Fig. 2 never reaches the experimentally determined toxic levels (steady state below 0.3 mM). To reduce the complexity of the model, all FAS-catalyzed reactions of the fatty acid biosynthesis were removed, since hexanoyl-CoA can now directly be produced from hexanoic acid.

Hexanoic acid is uptaken by diffusion and we must consider a limitation. From a bioengineering perspective, uptaken concentration must not exceed the cytotoxic concentration. Intracellular hexanoic acid needs to be converted intracellularly rapidly to hexanoyl-CoA to avoid toxicity caused by accumulation. We determined cytotoxicity of EC_50_ ≈ 1.2 mM in simple incubation assays (unpublished data by authors). This is achieved by heterologous expression of *Cannabis sativa AAE1*, encoding an acyl-activating enzyme for various short- and medium-chain fatty acids. It is lacking the typical AAE-specific peroxisome targeting sequence and is thus localized in the cytoplasm (Gagne et al. 2012). AAE1 activates carboxylic acids of the hexanoic acid through an adenylate intermediate that is occurring naturally in *S. cerevisiae*.

**Fig. 3:**
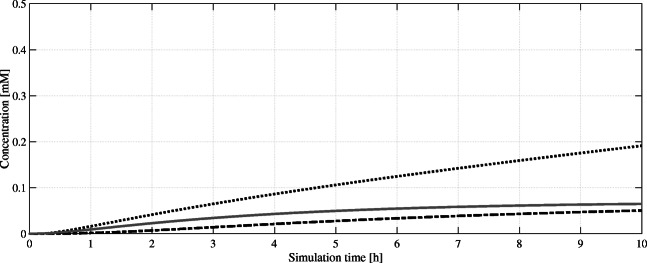
Simulated intracellular concentrations of olivetolic acid (OA, ), geranyl pyrophosphate (GPP, ), and (CBGA, ) over a simulation time of 10 h. After several genetic optimizations and in contrast to the accumulation of GPP, OA reaches a stable equilibrium concentration. This implies that it is the limiting intermediate in the subsequent formation of CBGA

Subsequently, the resulting hexanoyl-CoA is converted to olivetolic acid catalyzed by the heterologous enzymes olivetol synthase and olivetolic acid cyclase. Both enzymes are located in the cytosol, and the high metabolic activity in *S. cerevisiae* has been demonstrated. Besides olivetolic acid (OA), three other by-products occur as Gagne et al. (2012) showed: first, hexanoyl triacetic acid lactone (HTAL); second, pentyl diacetic acid lactone (PDAL); and last olivetol. The basis for this by-product formation is the hydrolysis of the common precursor hexanoyl-CoA and dodecane-tetraon-CoA (Do-Te-CoA). Due to promiscuity olivetol synthase catalyzes the reaction of Do-Te-CoA to mentioned compounds. The product ratios have been experimentally verified by the authors to be 95 % olivetol and 5 % olivetolic acid with only trace amounts of HTAL and PDAL. Though initially low, olivetolic acid levels can be improved through bioengineering. Site-directed mutagenesis studies revealed a variant of OAC (Y27F) with 162 % relative activity compared with the wild-type enzyme (Yang et al. 2016). Further improvement in olivetolic acid production was achieved by a fusion of both enzymes using a linker method and the addition of further copies of these linked enzymes (Luo et al. 2019). Both enzymes (OAC and OLS) were considered one reaction in our model to simplify due to close proximity of both enzymes and direct transfer of the substrate between both (Fig. 3).

### GPP production by the mevalonate pathway

The production of geranyl diphosphate (GPP) by the mevalonate pathway bears significant potential for metabolic engineering approaches. It features several enzymes with remarkably low conversion rates and concludes in an unfavorable ratio of farnesyl diphosphate (FPP) to geranyl diphosphate (GPP). The initial Claisen condensation of two acetyl-CoA to acetoacetyl-CoA in a thiolase (encoded by *ERG10*) reaction, followed by the conversion to 3-hydroxy-3-methyl-glutaryl-CoA (HMG-CoA) are fast catalytic reactions. However, the following enzyme HMG-CoA reductase, catalyzing the formation of mevalonate, is especially slow and thus rate-limiting to the whole pathway(Burg and Espenshade 2011). Mevalonate is phosphorylated twice in succession, before the enzyme-bound intermediate is being decarboxylated to isopentenyl diphosphate (IPP). The following isomerization of IPP to dimethylallyl diphosphate (DMAPP) is again a slow conversion carried out by isopentenyl pyrophosphate isomerase (IDI1). The last step towards GPP is the 1′-4 coupling of IPP and DMAPP by the yeast farnesyl diphosphate synthase encoded by *ERG20*. The native enzyme shows a geranyltranstransferase activity besides the aforementioned dimethylallyltranstransferase activity and converts GPP to FPP using another IPP moiety. This is undesired for the heterologous cannabinoid pathway, as only GPP is accepted by subsequent prenyltransferase. Ignea et al. (2014) showed that using an *ERG20* double mutant (F96W-N127W), FPP synthesis was significantly reduced in favor of GPP synthesis, and monoterpene yields were drastically increased.

**Fig. 4:**
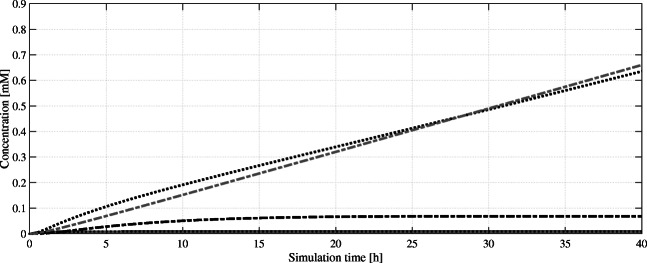
Simulated intracellular concentrations of mevalonate pathway intermediates over a simulation time of 40 h. While farnesyl pyrophosphate (FPP, ) and geranyl pyrophosphate (GPP, ) accumulate, cannabigerolic acid (CBGA, ) reaches a steady state while being used as substrate itself. Neither isopentenyl pyrophosphate (IPP, ) nor dimethylallyl pyrophosphate (DMAPP, ) are detectable in significant amounts as they get converted into GPP/FPP efficiently

To address the mevalonate production, Harker et al. (2003) suggested using an N-terminal truncated *Hevea brasiliensis* HMGR (tHMGR) version. This enzyme is missing its ER-membrane binding domain, which overall led to an 11-fold increased activity compared with the insoluble enzyme. Furthermore, other groups like Chen et al. (2012) and Ro et al. (2006) demonstrated that overexpression of *tHMGR* improved monoterpene production significantly. Like tHMGR, overexpressed IDI1 increased geraniol titers by 51 %, as documented by Zhao et al. (2016). In the published kinetic model, both overexpressed enzymes (tHMGR, IDI1) are represented by their native forms with higher V_max_ values. Also, the increased activity of *H. brasiliensis* HMGR through truncation and the modified ERG20 is implemented to simulate the GPP-optimized mevalonate pathway. A simulation of the model over a time period of 40 h reveals a near-complete utilization of IPP/DMAPP to form GPP and FPP (Fig. 4). Although GPP is continuously converted to CBGA, an excess still accumulates over time to about 0.65 mM in parallel with the unwanted FPP. This indicates that the GPP/FPP formation is too fast, and not all GPP can be converted to CBGA that can be explained either by too low olivetolic acid supply or non-sufficient upstream conversion to THCA.

### Cannabinoid biosynthesis and THCA production

After the supply of olivetolic acid and GPP, the last two enzymatic catalytic steps towards THCA are the biotransformation to CBGA by a prenyltransferase and the subsequent conversion to THCA by THCA synthase (THCAS). Our in silico model utilizes the mutated soluble prenyltransferase NphB performing at multitudes of its wild-type activity, as mentioned. The THCAS kinetics are likewise fitted to match current maximal activities achieved by in vivo experiments. These include a combination of the previously discussed variants N89Q/N499Q and H494C/R532C, without any overexpression.

**Fig. 5:**
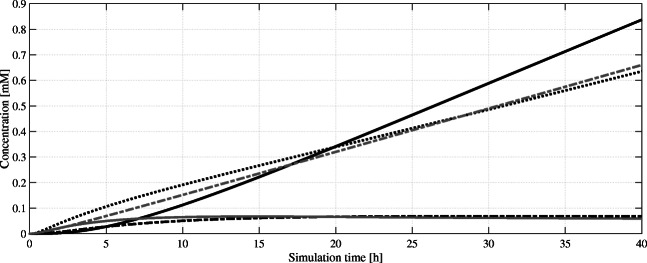
Simulated intracellular concentrations of cannabinoid pathway intermediates and product over a simulation time of 40 h. From the precursors geranyl pyrophosphate (GPP, ) and olivetolic acid (OA, ), cannabigerolic acid (CBGA, ) is formed, which itself is converted to the product Δ^9^-tetrahydrocannabinolic acid (THCA, ). The remaining farnesyl pyrophosphate (FPP, ) concentration can be seen as potential to increase GPP production in future attempts

Figure 5 depicts key intermediates as well as the THCA product formation in a complete pathway simulation over 40 h. At this time point, a comparable in vivo culture will reach the stationary phase after most of the available carbon sources are depleted. Since our model uses exponential phase kinetic parameter data, we stop our simulation here to preserve expressiveness. Furthermore, it is evident that several process parameters of an in vivo cultivation (biomass turnover, generation cycle, feeding of glucose, pO_2_, pH, etc.) cannot be accounted for by this given model; it is an ideal case and may not reflect real conditions. Considering CBGA biotransformation first, an accumulation of both OA and GPP can be observed whereby OA reaches a steady state after 10 h in silico (Fig. 4). Hence, in our model the tested NphB variant can match the rate of OA biosynthesis. The detected GPP accumulation can be interpreted as the potential to the formation of additional CBGA equivalents, once the olivetolic acid pool can support this. The generated CBGA is efficiently converted to THCA, again with a slight accumulation of substrate hinting towards somewhat balanced reaction rates of CBGA formation to CBGA usage. All in all, the model outputs a final concentration of 0.837 mM or 299.8 mg/L after 40 h.

**Fig. 6:**
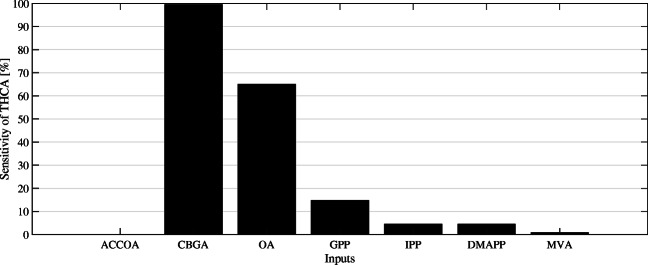
Sensitivity analysis of the output THCA concentration. The bars represent the relative magnitude of THCA concentration increase when one intermediate is varied over time. Acetyl-CoA (ACCOA), cannabigerolic acid (CBGA), olivetolic acid (OA), geranyl pyrophosphate (GPP), isopentenyl pyrophosphate (IPP), dimethylallyl pyrophosphate, and mevalonate (MVA) are chosen as input intermediates to be varied in the analysis

### Critical analysis of key enzymes, pathway bottlenecks, and potential for improvement

The suggested kinetic model comprises a collection of state-of-the-art bioengineering approaches for THCA biosynthesis and consolidates them into comprehensible determining factors. Several plots for various intermediates can be readily generated to evaluate the efficacy of a bioengineering strategy in distinct limitations. In this regard, the established model may serve as a basis for the decision and a helpful guide to rational pathway optimizations.

A product sensitivity analysis is a powerful utility within the MATLAB® software package to identify a target intermediate with a high impact on product formation. With THCA as the apparent product output for the analysis, eight key intermediates from mevalonate and olivetolic acid pathways were selected as inputs. The sensitivity shown in Fig. 6 can be interpreted as the positive change in THCA concentration over time at fluctuating input concentrations. The analysis suggests an increase in CBGA concentration to be most beneficial towards a higher THCA yield, closely followed by an increase of olivetolic acid. The engineering of corresponding enzymes, NphB and OAC, respectively, therefore should take priority over other enzymes. It must be noted that successful bioengineering of one enzyme to a higher catalytic rate, resulting in increased yields of the corresponding intermediate, immediately shifts other sensitivities. Heightened catalytic activity of NphB would, for instance, require the pathway to provide more olivetolic acid and GPP.

In this review, we employed the kinetic model to identify metabolic bottlenecks and to evaluate strategies to overcome them. We demonstrated that the wild-type *S. cerevisiae* host is exceptionally slow and inefficient at producing the GPP building block as we know from published data on monoterpene synthesis. To cover the demand in the pathway, a redesign of the key enzymes and transcription factors is essential, and we must mention the importance of enhanced HMGR, followed by ERG20, both important for delivering GPP. Furthermore, the native hexanoic acid production is still too low, and bioengineering the fatty acid biosynthesis towards a higher production can have taxing side effects through the omission of long-chain fatty acids. Again, feeding the essential hexanoic acid is a first solution to overcome metabolic bottlenecks but can definitively not been seen as an elegant synthetic biology solution. As the subsequent acyl activation must never be a bottleneck due to hexanoic acid toxicity, a cytosolic AAE from *C. sativa* should be recruited. Lastly, the sensitivity analysis shows promising results for bioengineering of NphB. This prenyltransferase has gone through remarkably successful rational protein designs to increase its activity and substrate acceptance significantly. Now, product specificity and efficacy must be improved in a disruptive approach to turn it from a truck motor into a jet engine.

## Bioengineering and process optimization

In recent years several processes were developed using *S. cerevisiae* as host organism to produce compounds like vanillin (Hansen et al. 2009), amorpha-4,11-diene (Ro et al. 2006), or artemisinic acid (Paddon et al. 2013), indicating that yeast has significant potential as an industrial platform host for large-scale production. To increase the THCA yield and to translate the lab-scale production to an industrial process scale, process parameters and cultivation conditions need to be optimized to keep costs down. In this review, we have described in detail the upstream process and did not pay attention to any downstream strategies. This was not the scope of this review but must always be addressed for a full cost budget plan.

No data regarding a successful scaled-up process have been published. Using an in silico yeast GMO model, we can predict a THCA titer of 299.8 mg L^−1^ after 40 h. This fermentation is based on an initial glucose concentration of 20 g L^−1^ and results in 0.015 g_THCA_ g_Glucose_ being produced, which is close to real production conditions. In our model we used glucose as a carbon source to produce THCA. Glucose is a cheap carbon source used in standard cultivation media for yeast, but alternative substrates based on molasses are an option too. As mentioned earlier, Luo et al. (2019) produced THCA and CBDA in *S. cerevisiae* based on galactose as the carbon source. This can only be accepted as a model case and is infeasible for industrial production due to being about 100-fold more expensive than glucose. However, the Crabtree effect is a challenge for production with high starting concentrations of glucose because cells are growing slower due to aerobic fermentation resulting in the production of ethanol (De Deken 1966). On the other hand, the produced ethanol can be used by the cells after diauxic shift due to glucose depletion. This reuse of ethanol was shown to increase amorpha-4,11-diene production in *S. cerevisiae* (Westfall et al. 2012).

Full biosynthesis of THCA in a whole-cell catalyst is most wanted, but feeding of substrate may boost production as well. Due to limited intracellular hexanoic acid delivery, feeding of HA is of high interest. As discussed above, toxicity must be addressed, why smart substrate supply by process control is essential. However, in terms of hexanoic acid feeding, it must be considered that growth of the yeast cells is negatively affected by hexanoic acid concentrations above 1 mM as we explained above and titers should therefore not exceed this concentration. The model, as we outlined here, predicts low CBGA titers even with hexanoic acid feeding. As a consequence, CBGA is limited, and the catalytic activity of used prenyltransferases needs to be improved to run THCA conversion at its maximum. This may raise the question of OA feeding. From its chemical and physical properties, OA is not well absorbed by yeast; it shows low chemical stability with high tendency to decarboxylation, and costs for synthesis are significantly higher than for hexanoic acid.

A further challenge is the functional expression of THCAS, especially when the copy number of THCAS integrations is increased. It was shown that reduction of the cultivation temperature from 30 °C to 20 °C leads to increased activity of the heterologously produced THCAS in *K. phaffii* (Taura et al. 2007). Zirpel et al. (2015) reported a 5-fold increased activity of THCAS by reducing the cultivation temperature from 25 °C to 15 °C through more efficient folding of the THCAS protein. A 20-fold increased activity of heterologously produced THCAS in *K. phaffii* was observed with co-overexpression of THCAS with the chaperone CNE1, FAD synthetase FAD1, and the transcription factor HAC1s (Zirpel et al. 2018a). These early findings explain very well that besides of genetic yeast optimization, smart bioengineering of process parameters will play an equal role in the future of cannabinoid biotechnology.

As discussed above, increasing titers of THCA may impact the vitality of the yeast cell, and toxic effects might limit the biotechnological production. A solution to detoxify cannabinoids and allow high yield production can be glycosylation of cannabinoids in order to make them water-soluble and to reduce cytotoxicity as demonstrated for vanillin (Hansen et al. 2009). Glycosylation strategies for cannabinoids have already been discussed, and the experimental proof was published recently (Hardman et al. 2014). These positive effects were described by Moehs et al. (1997) and Lim (2005), and the concept was successfully applied on an industrial level for heterologous vanillin biosynthesis in yeast.

## Conclusion

A systems biotechnology approach for high yield heterologous THCA biosynthesis is still in its infancy and must be characterized by low titers. Today, the question is not anymore what genes and biocatalysts must be used; the driving force in bioengineering 2.0 is to make kg and not mg to be competitive with plants. After careful analysis of metabolic bottlenecks in the heterologous THCA production, we can identify as critical the following:

1. Insufficient hexanoic acid formation in the fatty acid biosynthesis
2. Low acetyl-CoA precursor delivery to the hexanoic acid biosynthesis and the mevalonate pathway
3. Limiting catalytic activity of NphB or CsPT and THCAS
4. Insufficient ATP and NADPH regeneration
5. Ethanol production by Crabtree effect

From our calculations and experimental data, it is obvious that hexanoic acid production is a limiting step for the complete cannabinoid biosynthesis. Here, future metabolic work must resolve this bottleneck towards the delivery of olivetolic acid. By calculation of yeast performance to deliver a sufficient amount of olivetolic acid, a serious bottleneck remains unsolved. Hexanoic acid is at low concentrations and cannot cover the demand for sufficient olivetolic acid production. Only 5 % of HA is converted to OA, while 90 % is olivetol in practice. If further modifications like glycosylation are necessary, a third of THCA is converted according to Elshahawi et al. (2015) and Gachon et al. (2005) following simple rules of glycosylation. Taking into account that no delivery of HA as precursor will satisfy the demand of olivetolic acid for prenylation with GPP, feeding with synthetic OA with all its consequences is only a second option.

A combination of the kinetics provided by Chen et al. (2012) and Förster et al. (2003) are used for the throughput calculation of glycolysis (Förster et al. 2003; Sheng and Feng 2015). The reactions of acetyl-CoA to isopentenyl pyrophosphate and dimethylallyl pyrophosphate were modeled with the data and kinetics of Smallbone et al. (2013) and the geranyl pyrophosphate synthase with the data of Ku et al. (2005).

THCA production by a genetically engineered *S. cerevisiae* strain was successfully modeled, and a bioprocess was in silico designed that allows prediction of industrial applications. Modeled pathways and obtained data showed clearly that the supply of GPP and olivetolic acid from the primary pathways is limiting the THCA biosynthesis in yeast. Especially low concentration of hexanoic acid is critical for high yield production of THCA. This can be solved but must be considered the top priority for any metabolic engineering of the yeast. We have not discussed other aspects of rational and smart metabolic engineering like transcription factors, import and export of substrate, and THCA as final products, but these must be validated as well in future modeling approaches. With all limitations, *S cerevisiae* is still the best platform organism we have to produce heterologously cannabinoids, but it is obvious that a perfect running yeast will not compete with *C. sativa* L. varieties known for THC concentrations of 20 % and more. Plant extraction will stay for a long time the first choice for THC delivery. But the cannabis biotechnology has its unique niche for the production of so-called minor or rare cannabinoids, which are present at very low concentrations of less than 0.5 % in dried flowers. Smart bioengineering will be an attractive alternative to the cannabis plant.

## Data Availability

All code will be provided on request by the corresponding author OK. It can be run in a MATLAB version 9 software environment with an embedded Simbiology Toolbox.
